# Refractive changes of a new asymmetric intracorneal ring segment with variable thickness and base width: A 2D finite-element model

**DOI:** 10.1371/journal.pone.0257222

**Published:** 2021-09-15

**Authors:** Gonzalo García de Oteyza, Juan Álvarez de Toledo, Rafael I. Barraquer, Sabine Kling

**Affiliations:** 1 Clínica Oftalmológica García de Oteyza, Barcelona, Spain; 2 Escuela de Doctorado, Universidad Autónoma de Barcelona (UAB), Barcelona, Spain; 3 Centro de Oftalmología Barraquer, Barcelona, Spain; 4 Universitat Internacional de Catalunya (UIC), Barcelona, Spain; 5 Department of Information Technology and Electrical Engineering, OPTIC Team, Computer-assisted Applications in Medicine Group, ETH Zurich, Zurich, Switzerland; Universidad de Monterrey Division de Ciencias de la Salud, MEXICO

## Abstract

**Purpose:**

To evaluate the local geometric effects of a unilateral intrastromal ring segment with a combined variation of ring thickness and base width in a finite element simulation, and to compare it against the isolated effect of thickness or base width variation alone.

**Methods:**

A two-dimensional finite-element model of a transversely isotropic cornea was created assuming either axisymmetric stress or plane strain condition. The model geometry was composed of a three-layered corneal tissue (epithelium, anterior and posterior stroma) fixed at the limbus. The implantation of a triangular-shape asymmetric ring segment with varying ring thickness (150 to 300 μm) and base width (600 to 800 μm) was simulated. Also, changes induced by thickness or base width alone were studied and compared their combined effect in the asymmetric ring segment. Geometrical deformation of the simulated cornea and sagittal curvature were the main parameters of study.

**Results:**

Increasing ring thickness and base width along the arc of the asymmetric ring segment produced a more pronounced flattening in this part of the ring. The asymmetric design did find a good balance between maximizing corneal flattening at one end and minimizing it at the other end, compared to the isolated effect of ring thickness and width. Ring thickness was the most robust parameter in flattening both, the central and peripheral cornea.

**Conclusion:**

The finite-element model permitted a theoretical study of corneal deformation undergoing implantation of realistic and hypothetical ring geometries. Intracorneal asymmetric ring segments with varying thickness and base width can be a good alternative in corneas with asymmetric keratoconus phenotypes.

## Introduction

Among keratoconus (KC) treatments, intracorneal ring segments (ICRS) have widely demonstrated their efficacy in improving clinical parameters and reshaping the cornea [1]. Through the last decades, the indications, geometry, and materials of ICRS have changed, looking for better biocompatibility and results [2]. Today, the most frequently implanted ICRS for keratoconus are made of polymethyl methacrylate (PMMA), have a triangular shape, and are available in different thicknesses and arc lengths [3]. However, despite all the technological advances in diagnosis, surgical techniques and nomograms, poor and disappointing results with ICRS have been reported in some cases, forcing positional changes in second surgeries and even changing or explantation [4–6]. This could be explained because KC with asymmetric patterns (“snowman”, “duck”, and “irregular croissants”, with no concordance among topographic and comatic axes) are treated with symmetrical ring segments (same thickness and base width across all segment) leading to lack of concordance between the visual and tomographic results [7]. For this reason, ICRS of constant thickness and base width have been stated to be excellent astigmatic correctors in symmetric phenotypes but deficient in the control of coma and corneal irregularity in asymmetric patterns, which could be fundamental for the acquisition of excellent visual results [8]. This hypothesis has led the scientific community to investigate different ring segment design variations, including asymmetric segments [9–12]. A new type of ICRS (AJL Pro+ from AJL Ophthalmic) has been recently designed for its use in KC patients. These ring segments show variations along their arc length in terms of thickness and base width.

Corneal biomechanical models are useful not only to understand the cornea’s theoretical response to refractive surgery, but also to have a more accurate prediction of the postoperative results in real patients. Here again, the finite element models (FEM) have improved in refinement and precision from a single-membrane model with linear elastic [13] or viscoelastic [14] material properties, to hyperelastic orthotropic shell models that account for the tissue’s microstructure [15], as well as solid viscoelastic and nonlinear [16] corneal models up to patient-specific models [17]. Previous literature on the implantation of ICRS is limited. Initial studies concentrated on axisymmetric models to evaluate the effect of geometrical ICRS parameters on the induced refractive change [18]. More recently, three-dimensional models considering a generic [19] or patient-specific cornea [20] have been proposed. Inherent to all previous simulation studies is that, to the best of our knowledge, only overall changes in corneal curvature have been assessed and no localized changes in sagittal curvature have been reported so far. The latter would be particularly interesting because (i) most clinicians assess post-surgical changes by means of such curvature maps and (ii) keratoconus is an asymmetric disease and therefore surface homogeneity plays an important role in the refractive outcome.

The current study aims to develop a finite element model (FEM) to evaluate the theoretical local geometric effects of a new asymmetric intrastromal ring segment with varying ring thickness and base and compare the results with another ICRS in which base width and thickness were fixed.

## Methods

### Finite element modelling

A FEM was created in ANSYS software (Mechanical APDL, Release 19.2, Canonsburg, Pennsylvania, U.S.A.). Due to computational load and complexity, the implantation procedure was limited to 2D. The geometry was composed of a three-layered corneal tissue (epithelium, anterior stroma, posterior stroma) that was rigidly fixed at the limbus with the possibility to rotate. Anterior and posterior corneal radii were set to 7.8 and 6.4 mm, respectively, similar to the Gullstrand-LeGrand schematic eye (r_ant,Gull_ = 7.8 mm, r_post,Gull_ = 6.5 mm). Central corneal thickness was modelled to amount 550 μm to simulate standard tissue, and 450 μm to represent a keratoconic cornea. A quadrilateral mesh consisting of 385 elements of PLANE183 (8-node structural solid) was created and a transversely isotropic material model was assigned in spherical coordinates:

$$\left[\begin{array}{l} \varepsilon_{rr}\\ \varepsilon_{cc}\\ \varepsilon_{cc}\\ \varepsilon_{cc}\\ \varepsilon_{cr}\\ \varepsilon_{rc} \end{array}]=[\begin{array}{cccccc} \frac{1}{E_{r}} & -\frac{v_{cr}}{E_{c}} & -\frac{v_{cr}}{E_{c}} & 0 & 0 & 0\\ -\frac{v_{rc}}{E_{r}} & \frac{1}{E_{c}} & -\frac{v_{cc}}{E_{c}} & 0 & 0 & 0\\ -\frac{v_{rc}}{E_{r}} & -\frac{v_{cc}}{E_{c}} & \frac{1}{E_{c}} & \frac{1+v_{c}}{E_{c}} & 0 & 0\\ 0 & 0 & 0 & 0 & \frac{1}{2G_{cr}} & 0\\ 0 & 0 & 0 & 0 & 0 & \frac{1}{2G_{rc}}\\ 0 & 0 & 0 \end{array}]\cdot [\begin{array}{l} \sigma_{rr}\\ \sigma_{cc}\\ \sigma_{cc}\\ \sigma_{cc}\\ \sigma_{cr}\\ \sigma_{rc} \end{array}\right]$$ (Eq 1)

**Fig 1:**
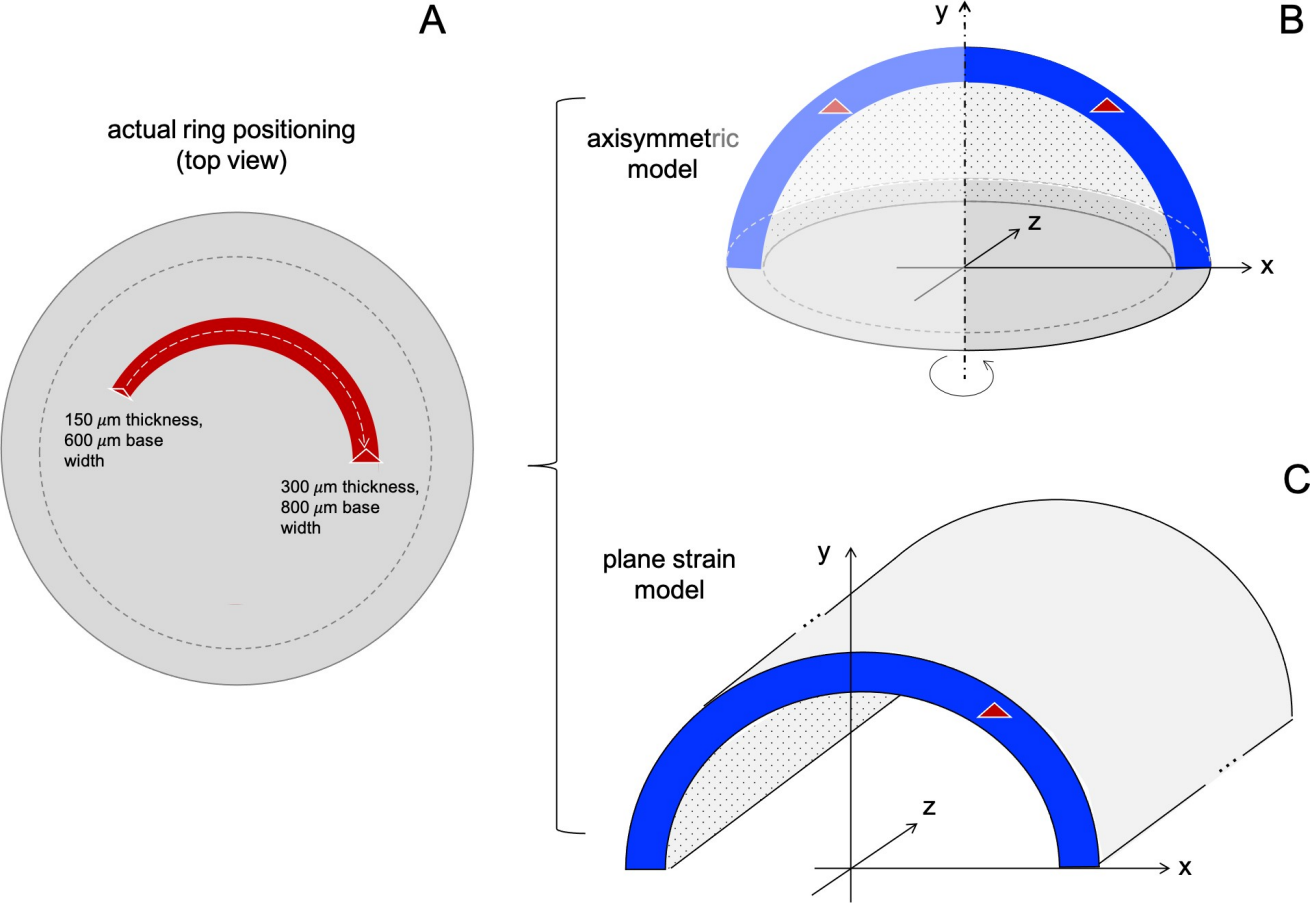
Schematic representation of the models used in this study. (A) Top view of the position of the ring into the cornea showing the variance of the thickness and base width. (B) Axisymmetric model where the cornea can be interpreted as a hemisphere symmetric across the y-axis. (C) Plane-strain model in which the cornea is considered infinitely extended along the z-axis and can be interpreted as a hemi-cylinder.

Where *ε*_rr_ is radial strain, *ε*_cc_ is circumferential strain, *ε*_rc_ is shear strain, *σ*_rr_ is radial stress, *σ*_cc_ is circumferential stress, *σ*_rc_ is shear stress, *υ*_cr_ and *υ*_cc_ is Poisson’s ratio in the two directions, *E*_r_ is radial elastic modulus, *E*_c_ is circumferential elastic modulus and *G*_rc_ is shear modulus. Given that a 2D model cannot fully describe the complexity of a 3D asymmetric ring (Fig 1A), two different models were investigated: In the first model, an axisymmetric stress condition was assumed, similar to previous literature [18,21]. Axisymmetry in this context implies that the geometry was considered symmetric about the axis going through the apex and optic nerve head (y-axis). Hence, this model assumes that a 360° ring with constant geometry is implanted, i.e. the cornea can be interpreted as a hemisphere in this model (Fig 1B). In the second model, plane strain condition was assumed. Plane strain in this context implies that strain in the third dimension is considered negligible compared to the cross-sectional strains. This condition typically occurs in very thick bodies, i.e. the cornea can be interpreted as a half cylinder in this model (Fig 1C). To the best of our knowledge, plain strain models have not been used in the past for ICRS simulation. Please note that this condition was included here only for comparison and to explore the lower bound of expected refractive changes.

Material constants assigned to the epithelium, anterior stroma and posterior stroma can be found in Table 1. Mechanical parameters were chosen to reflect the fact that the anterior stroma is stiffer [22,23] than the posterior stroma and that tensile stiffness (along collagen fibers) is higher than compressional stiffness (along corneal thickness). The intraocular pressure (IOP) was simulated by applying a surface pressure of 15 mmHg to the posterior surface. A radial and circumferential pre-strain of 0.015 in the anterior and 0.010 in the posterior stroma was assigned to account for the deformation induced by applying an IOP of 15 mmHg. In the anterior cornea, the radial component was defined to be of compressive nature (i.e. negative), in the posterior cornea of tensile nature (i.e. positive). This agrees with our recent study [23] on optical coherence elastography, which revealed this two-layered strain distribution in corneal tissue subjected to IOP-based loading.

**Table 1:** Material constants applied in the finite element model.

epithelium		
E	100	Pa
υ_rc_	0.4	-
ρ	1000	kg/m^3
anterior stroma		
E_rr_	500	kPa
E_cc_	1	MPa
G_rc_	20	kPa
υ_cc_	0.34	-
υ_rc_	0.34	-
ρ	1062	kg/m^3^
posterior stroma		
E_rr_	400	kPa
E_cc_	800	kPa
G_rc_	16	kPa
υ_cc_	0.34	-
υ_rc_	-0.34	-
ρ	1062	kg/m^3^

*E*_r_ is radial elastic modulus, *E*_c_ is circumferential elastic modulus, *G*_rc_ is shear modulus, *υ*_cr_ is Poisson’s ratio, *υ*_cc_ is Poisson’s ratio, ρ is density.

Previous literature confirmed that ICRS provoke a localized mechanical effect in the periphery with little impact on mechanical stress in the central cornea [19]. As a consequence, the induced geometrical changes can be considered independent on the underlying corneal shape. For this reason, we did not specifically simulate a keratoconic geometry in the current study, as the main interest was to compare different ring designs on their flattening abilities.

**Fig 2:**
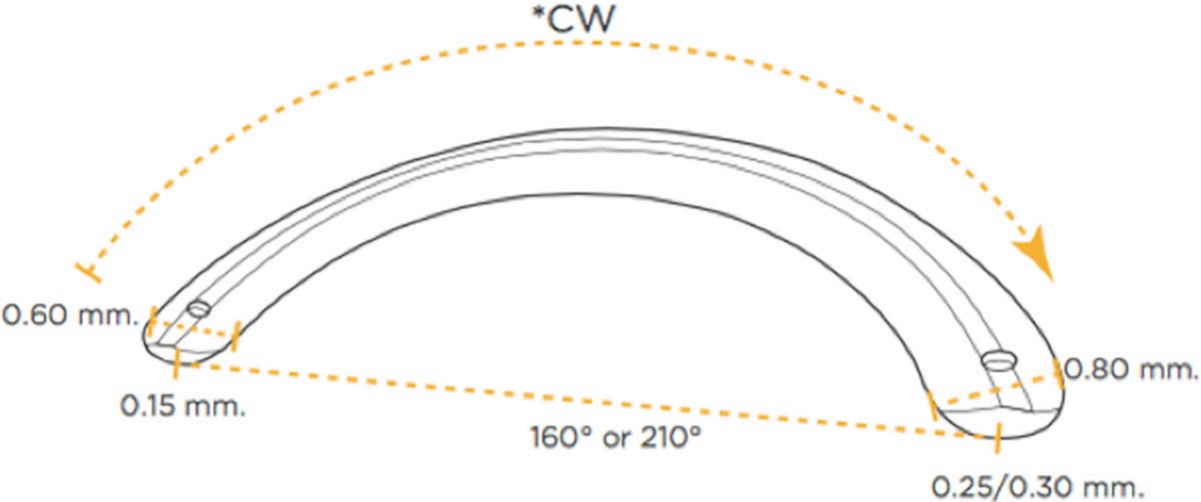
Geometry of the asymmetric ICRS.

The ring segment considered in the simulation is the asymmetrical ICRS fabricated by AJL (AJL Ophtalmic S.A., Vitoria, Álava, Spain) that spans over an arc of 160° and has a triangular cross-section. Its thickness increases from 150 to 300 μm and its base width from 600 to 800 μm from one end to the other (Fig 2). For the *in silico* implantation, first a stromal tunnel was created by modeling an area of the size 800 x 30 μm in 70% depth of the cornea that could either be meshed with stromal elements for the simulation of pre-op cornea, or be left free to represent the corneal tunnel for subsequent ring implantation. Next, the nodes at the inside of the tunnel were displaced according to the cross-section of the ring at a given angular position. For the accommodation of larger ring cross-sections, the tunnel was slightly expanded towards the inner edge given that the material at this location experienced the highest tensile load in radial direction. It was assumed that subjecting corneal tissue to a tensile stress oriented orthogonally to the fiber orientation would facilitate tissue rupture and expand the stromal tunnel during implantation surgery in clinics. After the deformation was imposed on the nodes, a rigid body was created out of the nodes forming the outer border of the ring segment. A rigid body approximates the fact that the material of the ICRS is much stiffer than the cornea. Finally, the nodal displacement constraints in axial direction were removed such that the ring could find its equilibrium within the cornea. The simulation was repeated for different angular positions along the ring segment. Furthermore, in order to evaluate the impact of thickness and base variation, the two parameters were changed independently.

### Curvature analysis

Coordinates of the deformed geometry were exported from ANSYS and read into Matlab (version R2017b, The MathWorks Inc, Natick, MA, USA) in order to compute sagittal curvature and best-fit sphere in the undeformed and deformed corneas, as well as the changes induced by the ring segment.

## Results

**Fig 3:**
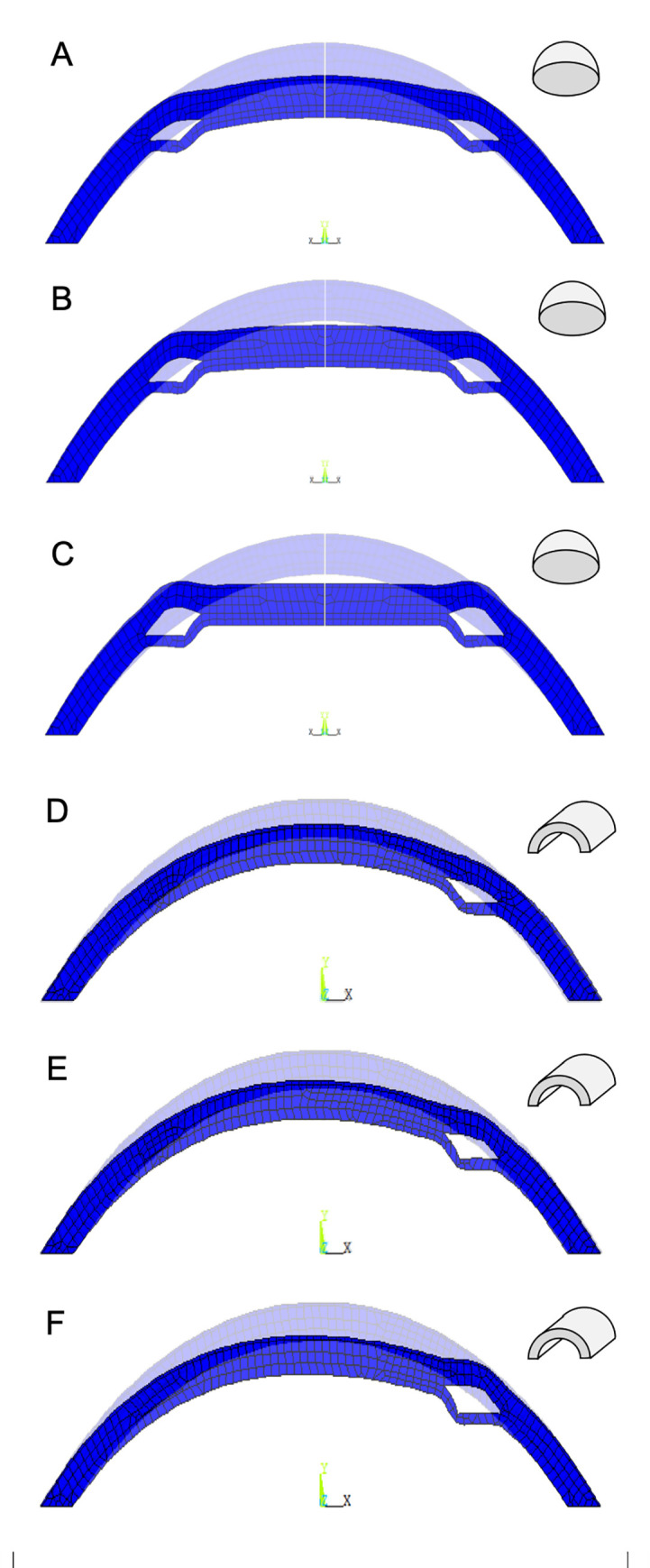
Deformed corneal geometry after implantation at different angular positions. The results are at (A,D) 0°, (B,E) 80° and (C,F) 160° in the axisymmetric (A-C) and plane-strain (D-F) simulation.

The deformed corneal shape resulting from the ring implantation at angular positions of 0° (small end), 80° and 160° (large end) is presented in Fig 3. As expected, towards the larger end of the ring segment a more pronounced flattening effect is observed. This observation is true for both, the axisymmetric and the plane strain model.

### Axisymmetric model

**Fig 4:**
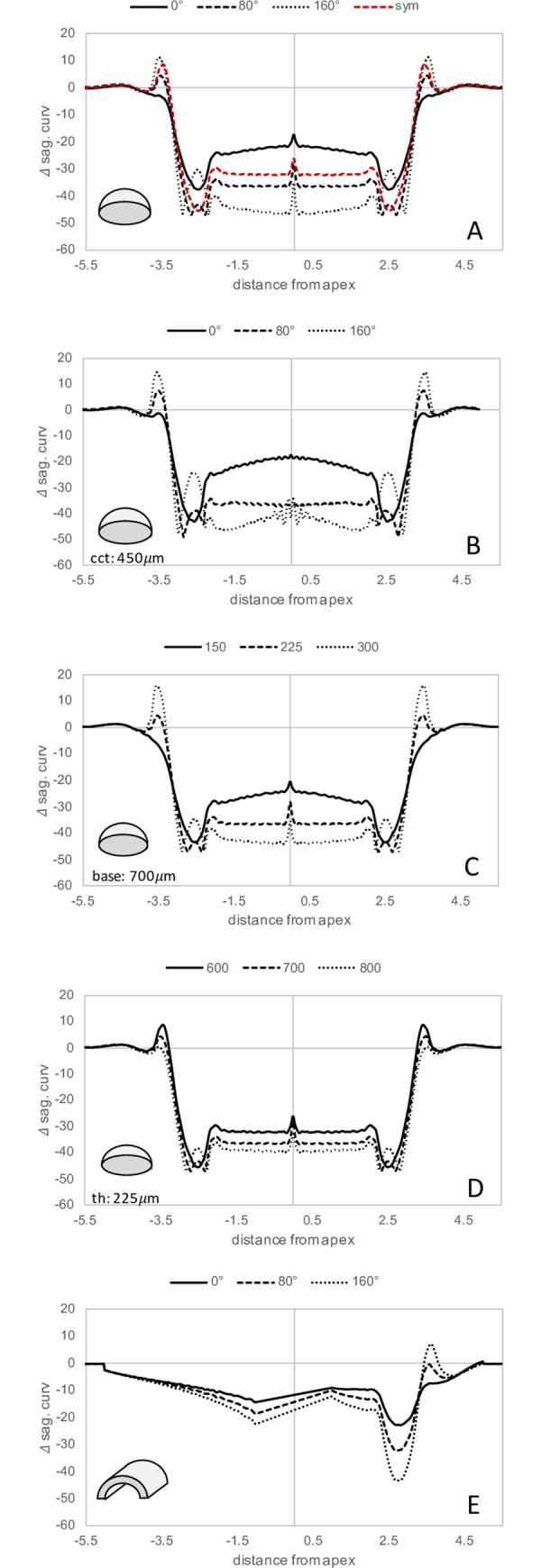
Change in sagittal curvature in the axisymmetric model with thickness and base variation of the ICRS. The results are presented in a (A) standard and (B) thin cornea, (C) with thickness variation only, (D) with base variation only, and in the plane-strain model (E) with thickness and base variation of the ICRS. The red dashed line in (A) represents a typical symmetric ICRS design with the mean thickness of the asymmetric ICRS.

The sagittal curvature changes induced by different ring segments are presented in Fig 4A–4C. The asymmetric ICRS with both, thickness and base width variation (Panel A, black lines) consistently induced peripheral applanation at all angular locations of the ring. As expected, the thinner and shallower end introduced less flattening (max -37.7 D) than the thicker and wider end (max -47.0 D). Also, the central flattening effect was more pronounced in the thicker and wider part of the ring segment. Symmetric ICRS currently used in clinics, typically have a base width of 600 μm and a constant thickness in the range of 150 to 350 μm. For comparison, its effect for a medium thickness of 225 μm (same as at 80° in the asymmetric ICRS) is plotted in red. Note here that these refractive changes occur at all angular positions of the symmetric ICRS.

To evaluate the effect of corneal thinning in combination with keratoconus, Panel B shows the induced refractive changes in an overall 18% thinner cornea (450 instead of 550μm). Refractive changes were nearly the same as with normal cornea thickness, only the most central applanation was approx. 2D lower in the tinner cornea.

In the hypothetical case that the base width was fixed to 700 μm and thickness allowed to vary from one end to the other, again the highest thickness produced the strongest flattening, while slightly less difference across the central optical zone of 4mm diameter between the two ends was observed (Δ = 17.0D) compared to the asymmetric ICRS in which the base was varied in addition (Δ = 22.4D). Moreover, with the lowest thickness a more pronounced central flattening (-24.7 D versus -21.7 D) was observed when the base was kept constant than when it was decreased as well (Panel C). Consequently, this effect was opposite with higher thickness where a weaker central flattening (-43.9 D versus -46.7 D) was observed when the ring width was kept constant.

In the hypothetical case that thickness was fixed to 225 μm and the base width allowed to vary from one end to the other (Panel D), a wider base produced the strongest peripheral (max -47.4 D) and central flattening (-39.8 D) at this ring thickness. Central curvature changes were more affected by the ICRS geometry than peripheral curvature changes. Overall, the difference across the central optical zone of 4mm diameter between the two ends was substantially smaller (Δ = 3.1D).

**Fig 5:**
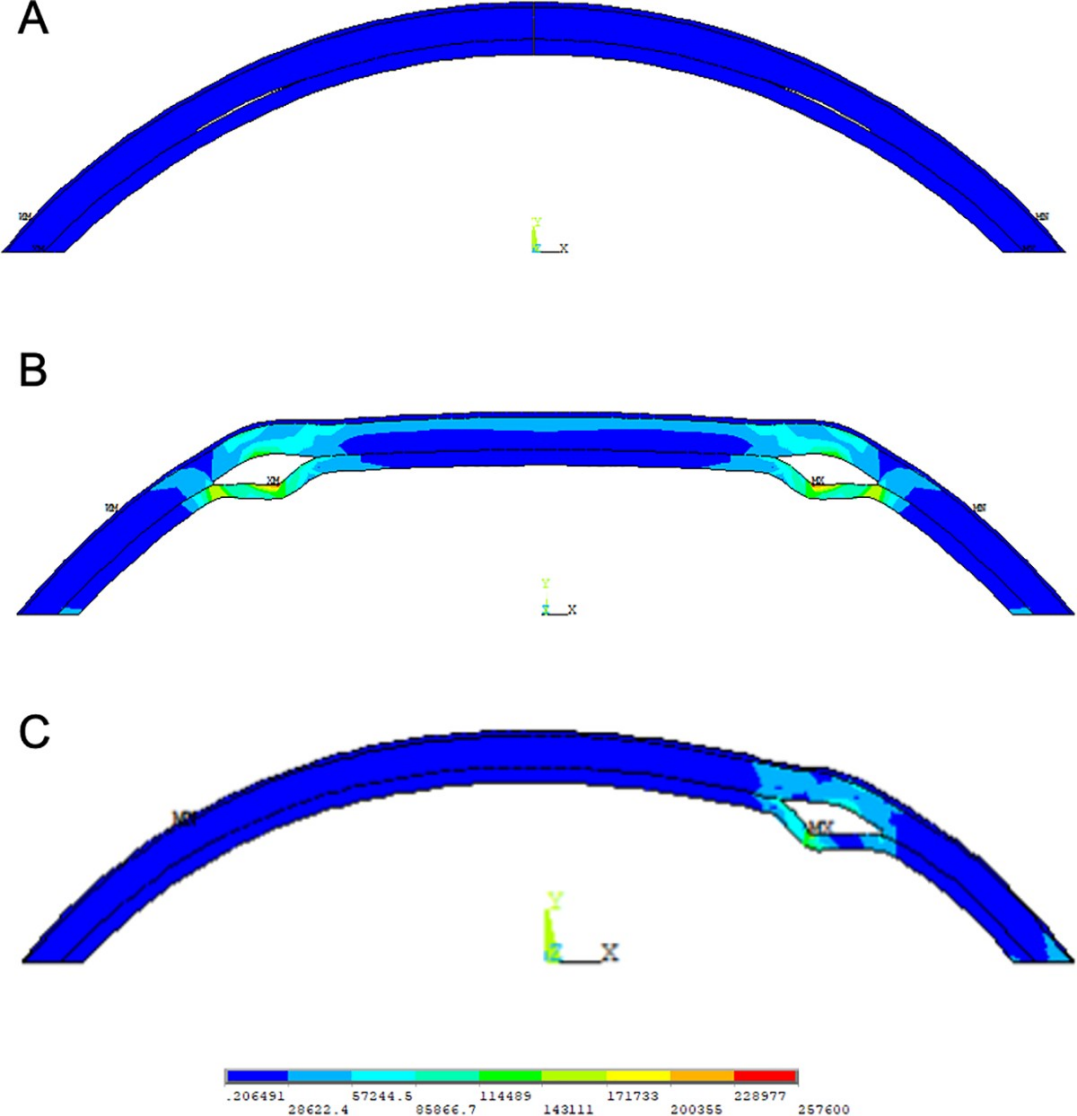
Von Mises stress distribution in the cornea. The results are shown after: IOP application (A), ICRS implantation with a cross-section corresponding to 80° of the asymmetric design in the axisymmetric (B) and plane-strain (C) FE-model.

Fig 5 presents the von Mises stress distribution resulting after ICRS implantation. Largest stress was induced in the direct vicinity of the implant and only minor (axisymmetric model) to negligible (plane strain model) stresses were present in the central cornea.

### Plain strain model

The sagittal curvature changes induced by an ICRS segment with both, thickness and base width variation ring are presented in Fig 4E. Similar to the axisymmetric model, the thinner and shallower end of the ring induced less peripheral (-22.9D) and central (-9.0D) flattening than the thicker and wider end (-43.5D and 12.1D, respectively). Due to difficulties in calculating sagittal curvature in the central zone (< 1mm radial distance from the apex) in this model, we interpolated this region. The refractive change was highest in the direct vicinity of the ICRS and gradually decreased towards the other peripheral side where no ICRS was inserted. Peripheral curvature changes were more related to ICRS dimension than central curvature changes. In fact, central refractive changes amounted approx. half of the changes predicted with the axisymmetric model for the same ICRS. Overall, the difference across the central optical zone of 4mm diameter between the two ends was Δ = 7.9D.

## Discussion

The comparison of an asymmetric ICRS in which thickness and base width vary along its arc length, with another ICRS in which only one parameter is modified, permitted to disentangle the effect of thickness and base width variation. We present two versions of a finite element model that describe the geometrical change of a generic cornea after implanting ICRSs of different geometries. Here we expect the axisymmetric model representing a 360° full ring to define the upper maximal possible correction and the plane strain model the lower minimal possible correction. Indeed, when looking at a single cross-section, corneal flattening induced by a bilateral ICRS (axisymmetric model) was nearly the additive effect of two unilateral ICRSs (plane strain model). This indicates that both models agree in their predictions.

Previous reports based on finite element models concluded important facts that afterwards were visible clinically. Firstly, an ICRS with triangular cross-section is the most effective geometry to treat KC and gets the best results regarding keratometry and refraction reduction [18,24]. Secondly, thickness has a linear relationship with refractive change; the thicker the ICRS is, the greater the refraction is lowered. And finally, smaller optical zones showed a more pronounced flattening effect. Those results proved in theoretical models were confirmed in real patients [8,18,25–27]. Another essential information offered by theoretical models is the impact of biomechanical properties of different regions in the corneal tissue. Fleming et al. only considered the anterior surface as responsible for the full impact of ICRS in reducing refraction [28]. However, recent analyses [18] have proven that, although the anterior corneal surface is the main location of the corneal refractive reduction (75.4%), it is not the only contributor. The axial displacement of the corneal apex also contributes to reduce the ocular refractive error by almost 13.4%. On the other side, changes in the posterior corneal surface increased corneal refraction by 12.3%.

Until now, all those theoretical models studied the effect of symmetric ICRS in normal corneas and KC. Our study is the first of this kind to approximate the refractive changes after the implantation of an asymmetric ICRS with variable thickness and base width. These rings are applied because outcomes after symmetric ICRS implantation are often inaccurate. Despite the most advanced technological diagnostic tools, refined nomograms of implantation, and the use of the latest femtosecond lasers during surgery, in some occasions the visual outcome is poor and unpredictable. It is speculated the reason for this is that ICRS do not address the asymmetry present in the large majority of KC corneas. In consequence, it has been suggested to treat KC not only depending on maximal keratometry or minimal pachymetry, but also regarding its phenotype. Alfonso created a new classification of KC orientated to phenotypic aspects of ectasia [29]. In this classification, the main parameters to describe KC are: location of the cone (central or paracentral); the relationship between refractive, topographic, and comatic axes; astigmatism; and symmetry. Thus, we can find symmetric KC known as “croissant”, “bow-tie” and “nipple” and also asymmetric phenotypes as “duck” and “snowman”. Asymmetric phenotypes have half of the ectasia more curved and elevated than the other, which provokes a higher coma aberration. One hypothesis is that poor results may be explained by the implantation of symmetrical ICRS into asymmetric ectasias and that asymmetric phenotypes could be better regularized with an asymmetric ICRS.

In the current study, we found that the asymmetric ICRS design did find a good balance between maximizing flattening at the more voluminous part of the ring, and limiting the flattening towards the less voluminous part. In particular, with respect to keratoconus treatment, where the aim lies in inducing a localized flattening in the cone region, the asymmetric ICRS is expected to be advantageous: First, it permits to provoke a more remarkable refractive change near the region of the cone. Second, by varying base width in addition to ring thickness, the refractive change responds to the different degree of required flattening at different angular positions. As a result, this type of ICRS is expected to produce a less distorted central optical zone. It should be maximally effective, positioning the higher and thicker part of the ICRS into the steepest area of the cone.

According to our analyses, the asymmetric ICRS locally flattens the ectasia between -21.5 D at the thinner and shallower end and -46.7 D at the thicker and wider end. Previously, ICRS induced refractive changes in finite element simulation have not been analyzed by means of sagittal curvature changes, but rather by changes in best fit sphere. For better comparison with those studies, the observed changes induced by the asymmetric ICRS correspond to a change in best fit sphere between -13.8D and -18.9D in the axisymmetric model, and between -12.8D and -14.0D in the plane strain model, over a central 10mm diameter optical zone. Studies in which symmetric ICRS were analyzed reported outcomes between + 4.09 D to -17.1 D [13]. Hence, the current model agrees with previous literature. Observed differences in the range of curvature changes between those models may be attributed to a different material model and a different way of ring insertion. Differences are more considerable, if we compare them to clinical results reported in the literature (-1 D to -6 D) [30,31]. Those differences may arise from the assumed input biomechanical properties that might not fully capture the mechanical characteristics of the tissue, as well as from the geometric approximations made in the model. It is a limitation of the presented model that it overestimates the effect of refractive changes. However, this effect has already been observed in earlier finite element simulation studies addressing ICRS surgery.^20^ Something that needs to be considered in this context is the epithelium’s ability to continuous remodeling. The fact that with time, it adapts its shape to inhomogeneities in corneal surface has not been accounted for in those models. Also, sagittal curvature is a very sensitive instrument to analyze localized geometrical changes and thus it will be greatly affected by the epithelium’s or Bowman’s layer response [32]. Therefore, the value of the current simulation study lies in the comparison of refractive changes that are produced by different geometrical parameters of the ICRS, rather than on the absolutely achieved refractive change. It is also important to emphasize that the aim of this simulation was to understand the functionality of different ICRS geometries rather than create a clinical predictive model. A further limitation is that the effect of keratoconic tissue has only been considered in a model with an overall thinner corneal tissue. While the expected potential of an asymmetric ICRS lies in the treatment of asymmetric keratoconus patterns, the axisymmetric model would have only allowed the simulation of a central symmetric keratoconus, while with the plane strain model a conical deformation could not be simulated at all. Given that we could confirm prior observations according to which ICRS-induced stress is mostly limited to the direct vicinity of the implant (Fig 5), we expect the overall thin cornea model being a representative condition that demonstrates the efficacy of an ICRS in keratoconic tissue. Accordingly, reduced corneal thickness in the conical region should hardly affect the refractive correction.

When comparing with a hypothetical ICRS where only one parameter, either thickness or base width is allowed to change, we can understand each parameter’s strength and importance. Thickness has shown to be the most robust parameter in flattening, both the periphery and the central cornea with on average $\frac{\Delta_{dioptre}}{\Delta_{thickness}}=0.13\;\frac{D}{\mu m}$ compared to base width, with on average $\frac{\Delta_{dioptre}}{\Delta_{width}}=0.04\;\frac{D}{\mu m}$. Our model suggests that combining the variability of ICRS thickness and base width concentrates flattening towards the larger end likely better preserves corneal surface homogeneity than symmetric ICRS in decentralized keratoconic eyes. We believe this outcome is a promising argument to further evaluate the clinical potential of an asymmetric ICRS in an asymmetric KC.

With respect to peripheral flattening we found that ring thickness dominates over base width. The higher the ring thickness is, the stronger is the achieved peripheral flattening. But at the same time, a more abrupt change in corneal geometry is induced resulting in substantial steepening in the surrounding of the ring (exterior to the optical zone).

Both, the results obtained from the axisymmetric and the plane strain model are not fully translatable to clinical application, because simulations either assume a 360° symmetric ring segment, or a cylindrical cornea. In fact, this study aimed not to present a model for clinical use since it cannot accurately reflect the corneal response to the implantation of the intracorneal segments. Many factors may influence the corneal response to the implantation of ICRS. Among them, scleral shape is one of the most important. It has been shown that the asymmetry in the scleral shape may influence the shape of the corneal periphery and therefore impact final astigmatism [33]. This evidence becomes even stronger when referring to keratoconus patients since anterior corneal curvature parameters are associated with the level of scleral asymmetry. As a rule, the more asymmetric a sclera may be, the higher curvature may be found in the anterior cornea [34]. Nonetheless, these models are complementary and allowed to investigate the differential effect of individual ring parameters on the produced refractive outcome with an asymmetric ICRS.

Still, future research investigating the interaction of the asymmetric ICRS with a 3D corneal geometry is required to refine the prediction of post-op refractive changes in patients, and to make the simulations patient-specific particularly in cases with decentralized, local alterations of corneal shape such as in keratoconus.

To sum up, according to the model, asymmetric ICRS with variable thickness and base width could be a good alternative for treating asymmetric KC phenotypes. This model allows us to conclude that combining two variable parameters effectively reduce corneal curvature and at the same time address the decentralization of the underlying degenerative pathology. This model permits a more objective analysis of how different geometrical parameters of ICRS affect the refractive outcome than a clinical study which necessarily has to deal with more confounding factors.
