# Correction: Refractive changes of a new asymmetric intracorneal ring segment with variable thickness and base width: A 2D finite-element model

**DOI:** 10.1371/journal.pone.0306652

**Published:** 2024-07-01

**Authors:** Gonzalo García de Oteyza, Juan Álvarez de Toledo, Rafael I. Barraquer, Sabine Kling

There are errors in the Author Contributions. The correct contributions are:

**Conceptualization:** Gonzalo García de Oteyza.
**Data curation:** Sabine Kling.
**Formal analysis:** Sabine Kling.
**Investigation:** Gonzalo García de Oteyza, Sabine Kling.
**Methodology:** Gonzalo García de Oteyza, Sabine Kling.
**Software:** Sabine Kling.
**Supervision:** Gonzalo García de Oteyza, Juan Álvarez de Toledo, Rafael I. Barraquer, Sabine Kling.
**Validation:** Gonzalo García de Oteyza, Juan Álvarez de Toledo, Rafael I. Barraquer.
**Visualization:** Gonzalo García de Oteyza, Juan Álvarez de Toledo, Rafael I. Barraquer, Sabine Kling.
**Writing–original draft:** Gonzalo García de Oteyza, Sabine Kling.
**Writing–review & editing:** Gonzalo García de Oteyza.
